# Busulfan pharmacokinetics in adults – a real-world evaluation of *intra*-individual variability and the impact of obesity and deferasirox

**DOI:** 10.1038/s41409-026-02833-0

**Published:** 2026-04-14

**Authors:** Claudia Langebrake, Eva-Maria A. Wansing, Dietlinde Janson, Christine Wolschke, Catherina Lueck, Francis Ayuk, Nicolaus M. Kröger, Adrin Dadkhah

**Affiliations:** 1 University Medical Center Hamburg-Eppendorf, Hospital Pharmacy, Hamburg, Germany; 2 University Medical Center Hamburg-Eppendorf, Department of Stem Cell Transplantation, Hamburg, Germany

**Keywords:** Translational research, Preclinical research

## Abstract

To avoid under- or overexposure caused by a high variability of busulfan’s pharmacokinetic parameters, therapeutic drug monitoring (TDM) is highly recommended. Nevertheless, it is not widely used in European transplant centers. We retrospectively analyzed real-world busulfan TDM data in adults in our center between 9/2016 and 11/2024 with special focus on overweight/obesity and comedication with deferasirox. Overall, 287 patients (161 male/126 female), median age 59.6 years (19.4–77.7), were included. Median calculated AUC after the first TDM was 17.1 mg*h/L with high interindividual variability (range: 9.6–37.9). Target attainment without TDM would only have been achieved in 59 patients (20.6%) using the standard dose, even if adjusted for overweight/obesity and comedication with deferasirox. Busulfan clearance (CL) was significantly reduced in patients receiving deferasirox (0.13 L/kg_dosing weight_ [0.07–0.22] vs 0.20 [0.12–0.33], *p* < 0.001), while no influence of body weight was detectable. Overall, there were no *Intra*-individual changes in both CL (−0.65% [−41.72–57.79]) and V_d_ (−1.40% [−36.85–69.04) between doses. However, in one third of patients relevant deviations were observed, regardless of either body weight or comedication with deferasirox. In contrast to some published data, no general decrease in CL over time was observed.

## Introduction

The alkylating agent busulfan (Bu) is often used as part of a conditioning therapy prior to an allogeneic hematopoietic stem cell transplant (allo-HSCT). It is known that Bu exhibits high variability between dose and exposure (area under the curve, AUC), even when administered intravenously [1].

Because of its narrow therapeutic index, over- or underexposure results in either toxic adverse effects and increased transplantation-related mortality or higher rates of graft rejection or relapse rates [2–7]. For this reason, about a decade ago, it was recommended to personalize busulfan dosing using a patient-individual therapeutic drug monitoring (TDM), especially in children and patients receiving myeloablative conditioning regimens [8]. However, busulfan TDM is not widely established among European transplantation centers, despite wide use of busulfan [9]. To increase the level of knowledge about the practical implementation of busulfan TDM and to overcome potential barriers to its introduction, European recommendations have recently been published [10].

Busulfan is a small and highly lipophilic drug that crosses the blood-brain barrier, resulting in similar concentrations in plasma and cerebrospinal fluid. The volume of distribution (V_d_) ranges between 0.62 and 0.85 L/kg body weight (BW) in adult patients. It is extensively metabolized in the liver. The main step is spontaneous or glutathione-S-transferase (GST) derived conjugation with endogenous glutathione (GSH). Subsequent steps involve metabolism via the mercapturic acid pathway or dissociation and further oxidation steps or further binding with GSH [11]. Presumably, none of the metabolites contributes significantly to efficacy or toxicity. Typical clearance (CL) for intravenous busulfan in adults is 0.2 L/h/kg, with a high *inter*-individual (between different patients) variability in a range of up to 3-fold reported in large studies [1, 12, 13]. In patients with obesity, CL best correlates with adjusted ideal body weight (AIBW) [14]. Therefore, the use of AIBW is recommended for dose calculation in patients with a body mass index (BMI) of 25 kg/m² and above.

Known clinically relevant drug-drug interactions with busulfan exist among others with acetaminophen, metronidazole, azoles, phenytoin or deferasirox resulting in subtherapeutic or supratherapeutic exposures [15, 16].

Especially in children, significant but hardly predictable *intra*-individual variability of busulfan’s pharmacokinetic parameters has been reported, with an overall decrease of busulfan CL between doses resulting in a risk of busulfan overexposure [17–19]. Respective data for adults are sparsely available at present.

The aim of this study is to analyze real-world busulfan TDM data in adults with special focus on overweight/obesity, comedication with deferasirox and *intra*-individual changes of busulfan’s pharmacokinetic parameters.

## Methods

Pharmacokinetic data of adult patients treated with TDM-guided busulfan-based conditioning as part of standard care treatment prior to allo-HSCT in the Department of Stem Cell Transplantation at the University Medical Center Hamburg between September 2016 and November 2024 were retrospectively analyzed. All patients signed written informed consents for treatment. The study was conducted in accordance with the Declaration of Helsinki and approved by the Ethics Committee of the Hamburg Medical Association (2022-100964-BO-ff).

Busulfan was administered intravenously over 3 h with an initial dose of 3.2 mg/kg once daily. The basis for dose calculation was total body weight (TBW) for patients with a BMI ≤ 25 kg/m^2^, while AIBW25 – calculated as ideal body weight (IBW) plus 25% of the difference between total and IBW – was used for patients with BMI > 25 kg/m^2^. In the following, the respective reference weight is referred to as “dosing weight”. With this approach, we aimed for an average busulfan-AUC of 20 mg*h/L (±10%) with initial dosing. According to the international Interlaboratory Proficiency Testing Program, deviations of ±10% for busulfan plasma exposure and dose recommendation are considered accurate [20]. Depending on the respective conditioning protocol and the intensity of conditioning – myeloablative (MAC) or reduced intensity (RIC) conditioning – two to four consecutive dosages at consecutive days (usually within the week before transplantation, but at least 24 h before stem cell infusion) – were planned to achieve the desired cumulative target AUC between 40 and 80 mg*h/L. TDM was performed as part of standard of care on the first or second day of application and the busulfan dose was adjusted accordingly based on these results.

Busulfan plasma levels were drawn according to a local sampling schedule 5 min, 1, 2 and 3 h after the end of busulfan infusion, whereas the exact infusion and sampling times were documented. Quantification was performed at the Department of Legal Medicine at the University Medical Center Hamburg-Eppendorf using a validated gas chromatography with mass spectrometric (GC-MS) detection method. The GC-MS method used was validated according to the EMA guideline on validation of analytical procedures in 2014 and all parameters including accuracy, precision and robustness complied with the specifications. Subsequently, model-based calculation of the AUC was carried out in the hospital pharmacy by means of Bayesian prediction using pharmacokinetics software (MW-Pharm, Version 3.60). If the calculated AUC was not within a target range of ±10% of the target cumulative AUC, busulfan dose was adjusted accordingly. In cases of dose adjustments >25%, new busulfan concentration measurements and AUC calculations were carried out after the subsequent dose, and, if necessary, a repeated dose adjustment was made.

According to local standards, supportive treatment included levetiracetam for seizure prophylaxis in all patients and deferasirox during conditioning in patients with a serum ferritin of >1000 µg/L. Deferasirox was administered at standard doses according to the Summary of Product Characteristics dependent on dosage form, starting on the first day of conditioning until day +3. Other drugs known to interact with busulfan (e.g. acetaminophen, metronidazole, azoles) were avoided.

The statistical analysis was carried out using IBM SPSS Statistics software version 29. Discrete variables were expressed as counts (percentages), whereas continuous variables were expressed as median and range. For group comparisons of categorial variables, chi-square, or Fisher’s exact tests were used as appropriate. Continuous variables that were not normally distributed were analyzed using Mann–Whitney-U-Test. A *p*-value < 0.05 was defined as statistically significant.

## Results

Overall, 287 patients (161 male, 56.1%) with a median age of 59.6 years (19.4–77.7) were included. Basic characteristics of the patient cohort are shown in Table 1.

**Table 1:** Patient characteristics.

	Median	Range
Age [years]	59.6	19.41–77.69
Total body weight [kg]	78.0	46.5–176.0
Ideal body weight [kg]	68.7	37.7–98.2
Adjusted ideal body weight [kg]	71.6	44.4–117.7
Height [cm]	175	144–205
BMI [kg/m²]	25.29	15.56–54.07

	*n*	%
Male sex	161	56.1
BMI > 25 kg/m²	130	45.3
Deferasirox	99	34.5
Underlying disease		
AML	150	52.3
MF	76	26.5
MDS	38	13.2
Multiple Myeloma	9	3.1
Other	14	4.9
Donor type		
MRD	43	15.0
MUD	188	65.5
MMUD	32	11.1
Haplo	24	8.4
Stem cell source: PB	284	99.0

Initial dosing of busulfan was 3.2 mg/kg_dosing weight_ once daily, calculated on TBW in 157 patients (54.7%) with BMI ≤ 25 kg/m² and on AIBW25 in 130 patients (45.3%) with BMI > 25 kg/m². During conditioning, 99 patients (34.5%) received deferasirox, 60 of them with an initial dose reduction of busulfan to 75–80%. TDM was performed at least twice in 138 patients (48%).

**Fig. 1:**
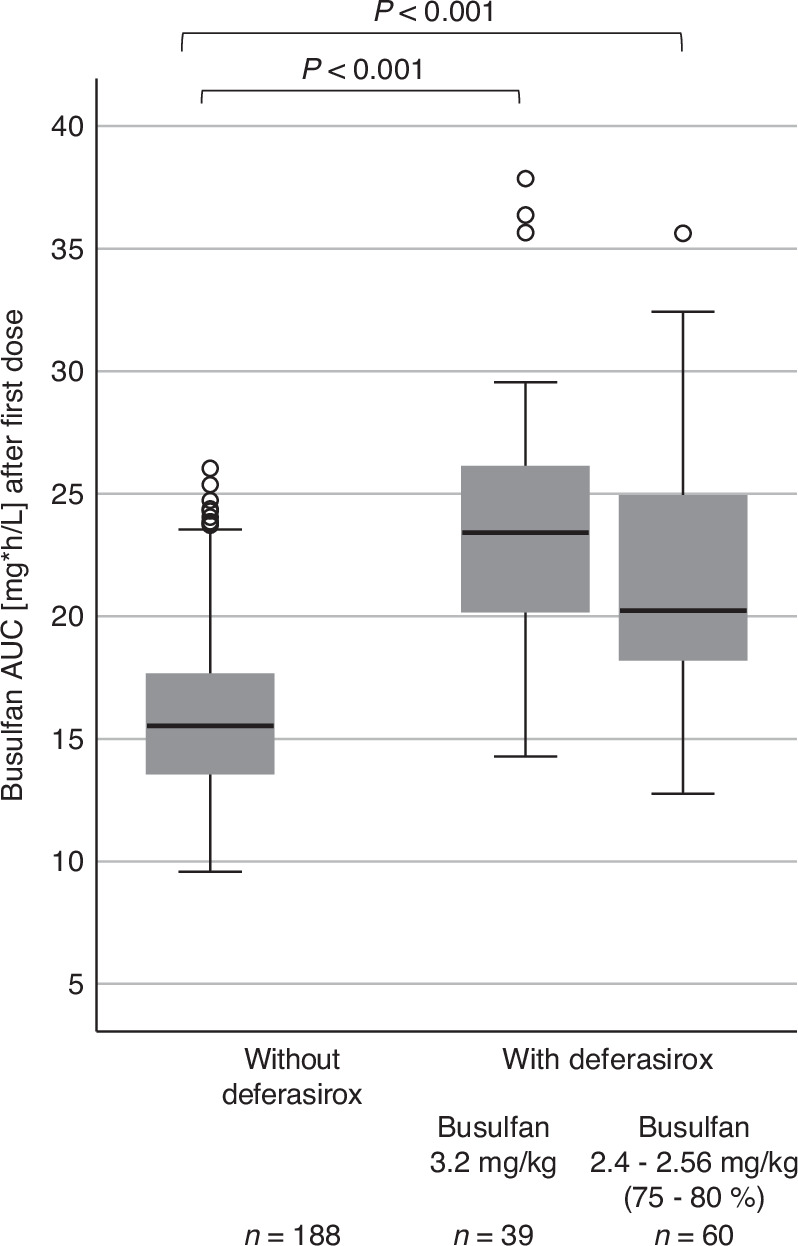
Busulfan exposure after the first dose in patients without and with deferasirox comedication, the latter divided into patients without and with initial busulfan dose reduction.

Median calculated AUC after the first TDM was 17.1 mg*h/L with high *inter*-individual variability in busulfan-exposure (range: 9.6–37.9). Overall, 59 patients (20.6%) reached the target-AUC (20 h*mg/L ± 10%) at the first TDM, while 165 patients (57.5%) needed a dose increase (93 patients of whom of more than 25% increase of the initial dose) and 63 patients (22.0%) a dose reduction (54 patients of whom of more than 25% reduction of the initial dose). There were no statistically significant differences in initial AUC or target attainment with regard to overweight/obesity (AUC: 17.5 [9.6–37.9] vs. 16.8 [9.7–32.4] mg*h/L). Concomitant deferasirox treatment resulted in significantly higher AUC at the first TDM (23.42 [14.26–37.86] vs. 15.54 [9.57–26.04, *p* < 0.001] mg*h/L), whereupon an initial dose reduction was implemented into the local conditioning protocols in 3/2023, resulting in more adequate median AUC-levels (20.25 mg*h (L [12.76–35.63]), as shown in Fig. 1.

**Fig. 2:**
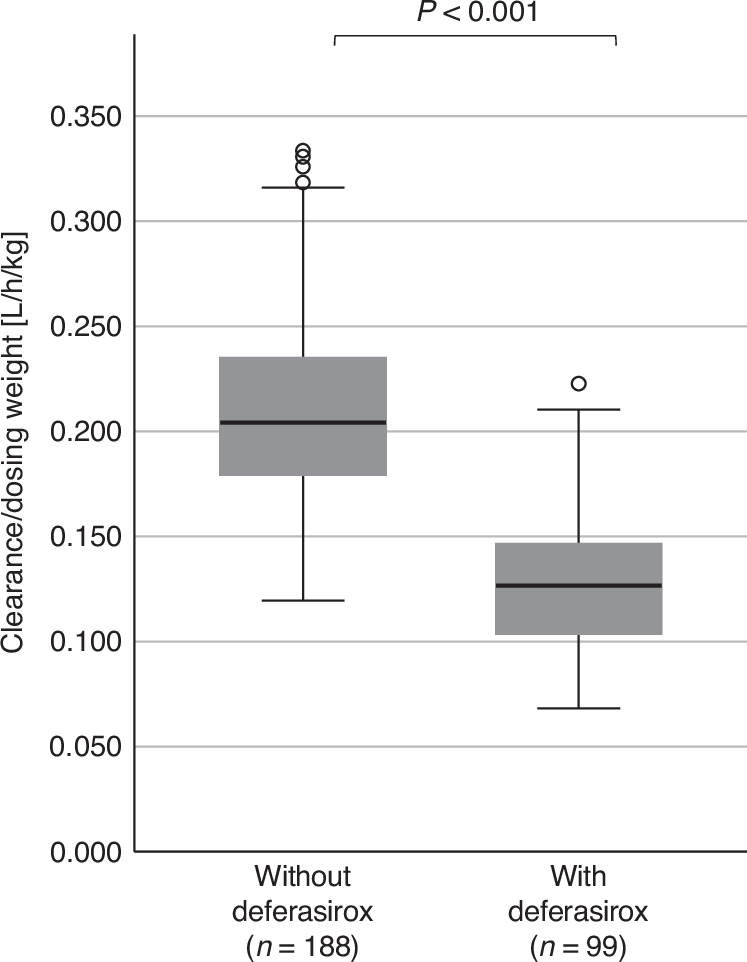
Busulfan clearance (CL) in patients with and without comedication with deferasirox.

Overall, busulfan CL was highly variable in the overall population with a median of 0.183 L/h/kg_dosing-weight_ and a range of 0.068–0.334, translating into a 4.89-fold *inter*-individual variability in CL. In patients receiving deferasirox, busulfan CL was 38% lower compared to patients without deferasirox comedication (0.127 L/kg_dosing weight_ [0.068–0.223 vs. 0.204 [0.119–0.334], *p* < 0.001); absolute difference −0.079 (95% CI: −0.088 to −0.069), translating into a 3.26 and 2.79-fold *inter*-individual variability, respectively (see Fig. 2). No influence of overweight/obesity on busulfan CL was detectable between patients with a BMI ≤ 25 kg/m² compared to patients with a BMI > 25 kg/m² (0.178 L/kg_dosing weight_ [0.068–0.334 vs. 0.186 [0.074–0.331], *p* < n.s.); absolute difference −0.008 (95% CI: −0.005 to 0.220).

**Fig. 3:**
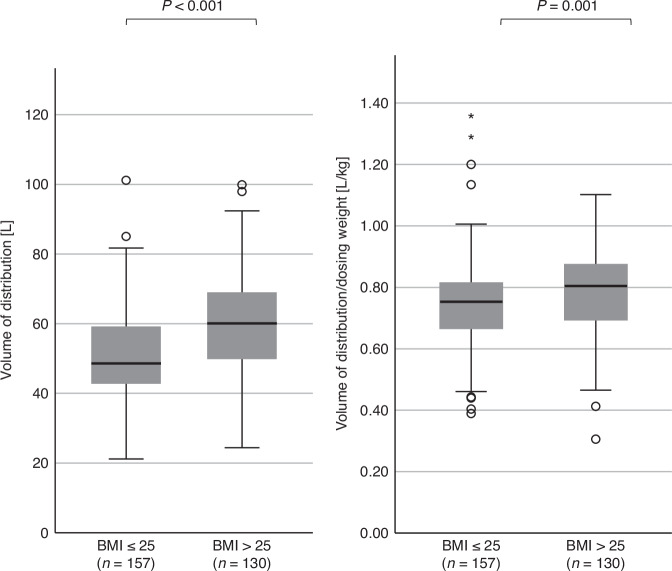
Volume of distribution (Vd) normal weight vs overweight patients (absolute Vd vs Vd/dosing weight).

In the overall population, median V_d_ was 54.41 L (range: 21.19–101.19 L) and 0.77 L/kg_dosing-weight_ (range: 0.31–1.36), with an *inter*-individual variability of 4.8- and 4.4-fold in V_d_, respectively, as shown in Fig. 3. Median V_d_ was significantly higher in overweight/obese patients (60.09 [24.4–99.9] vs 48.64 L [21.19–11.19]). When normalized to dosing weight, the difference still remained statistically significant, but the relative median difference accounted only for 7% (0.81 L/kg_dosing weight_ [0.31–1.10] vs 0.75 [0.39–1.36], *p* = 0.001).

**Fig. 4:**
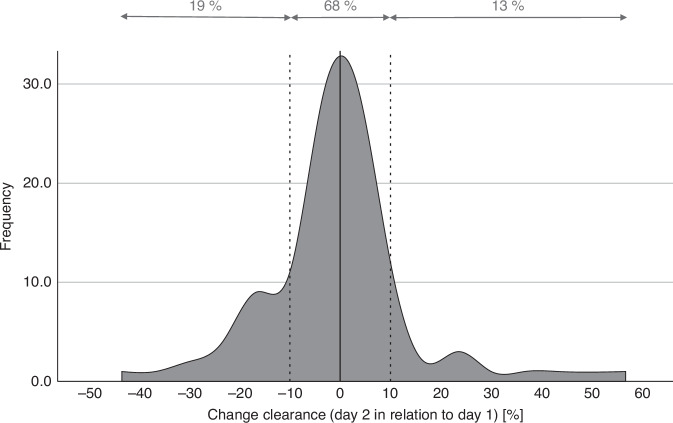
Relative change in busulfan CL at day 2 in relation to day 1.

*Intra*-individual changes of pharmacokinetic parameters between day 1 and day 2 of busulfan amounted to less than 1.5% in median: CL (−0.65% [−41.72–57.79]) or V_d_ (−1.40% [−36.85–69.04]). However, clinically relevant changes in CL of more than ±10% between doses occurred in 32% of patients, with 21/108 patients (19%) showing a decrease in CL of at least 10% and 14/108 patients (13%) an increase in CL of at least 10% (see Fig. 4).

**Fig. 5:**
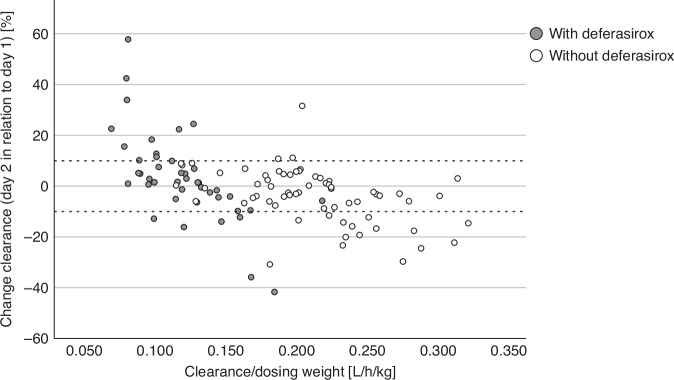
Relative between-dose change of busulfan CL at day 2 in relation to day 1 in patients with and without comedication with deferasirox.

These *intra*-individual between-dose changes in CL were similar in patients with and without overweight/obesity. In patients with or without comedication with deferasirox, median *intra*-individual changes in CL were below 4% in both groups (1.5% and 3.5%, respectively). However, we observed an increase in CL of more than 10% between day 1 and day 2 in more patients receiving deferasirox (26% vs 5%), and less frequently a decrease in CL of more than 10% (14% vs 23%) compared to patients not receiving deferasirox (see Fig. 5).

*Intra*-individual changes of V_d_ between doses 1 and 2 were similar in patients with and without overweight/obesity and with or without comedication with deferasirox.

## Discussion

We present real-world data on busulfan TDM in a large cohort of adult allo-HSCT patients. Target attainment without TDM would have been achieved in about 20% of patients using the standard dose, even when adjusted for overweight/obesity and comedication with deferasirox. The confirmed decrease in busulfan CL due to deferasirox can be compensated by an initial dose reduction of busulfan to 80%. High *intra*- and *inter*-individual variability of both CL and V_d_ was not different within subgroups of patients with either overweight/obesity or comedication with deferasirox. In contrast to some published data, no general decrease in CL after the first dose was observed, although relevant *intra*-individual (between-dose) changes of PK parameters frequently occur.

To avoid under- or overexposure caused by a high *inter*-individual variability of busulfan’s pharmacokinetic (PK) parameters, TDM – especially in children, for myeloablative conditioning or for regimes that were developed with PK-guided busulfan – is highly recommended [4, 8]. However, according to a European Survey, only 10–20% of transplant centers regularly perform busulfan TDM [9]. To overcome potential obstacles, a practical guidance on busulfan TDM including a step-by-step guide, was recently published and centers are encouraged to implement TDM for busulfan [10].

Using standard approved doses of busulfan, the majority of patients does not reach target exposure: about three out of five patients in our real-world analysis would have resulted in underexposure, while one out of five patients would have experienced overexposure, if no dose adjustment had been performed. This up to four-fold *inter*-individual variability between dose and exposure as well as the assymetrical distribution of busulfan exposure after the first dose are well known and is mainly explained by up to five-fold differences in drug CL [1, 12, 14, 21–23]. As expected, absolute V_d_ is higher in overweight/obese patients, but if normalized to dosing weight (AIBW in patients with BMI > 25 kg/m², otherwise TBW), these differences are less pronounced. This emphasizes the necessity of using adjusted weight measures for initial dosing, unless model-informed precision dosing (MIPD) is applied a priori [14, 21, 24, 25]. Consequently, median busulfan exposure after the first dose was similar in normal weight compared with overweight/obese patients. Furthermore, we could show, that the *inter*-individual variability of AUC, CL and V_d_ was not different between the two weight groups.

In a previous study, we demonstrated that concomitant deferasirox significantly reduces busulfan CL by one third [16]. We validated these findings in our current, even larger cohort of patients and could further show that an initial reduction to 75–80% of the body weight-adapted busulfan dose leads to an excellent median target AUC attainment. Notably, similar *inter*-patient variability between dose and exposure is observed in this patient group, necessitating dose reductions or increases in around 40% of the patients, respectively.

Data on *intra*-individual changes in busulfan CL over time exist mainly for children using four-times daily dosing of busulfan: Marsit et al. described that 80% of children experienced a decrease in busulfan CL, with a median change of −7.9% between day 1 and day 2, with high variability ranging from −48.5 to +44%. Over day 1 to day 3 mean CL significantly decreased by 15% [17]. In patients from 0.1 to 26 years, Bartelink et al. detected a 12% higher busulfan CL on the first day compared to subsequent days [18]. In a cohort of 58 pediatric and young adult patients, a decrease of busulfan CL of ~15% between first dose and steady-state dose PK sampling was detected by Long-Boyle et al. [19]. McCune et al. observed a slight decrease in busulfan CL over time and that children under four years of age have lower busulfan CL than adults [26].

Using a semi-mechanistic model, Langenhorst et al. observed an overall decrease of 7% in busulfan CL with a range of −82 to +44% in children and adults. Interestingly, there was an association between older age (>40 years) and a stronger time-dependent effect that increased by 4% per year of age. It has been suggested that this effect may be related to glutathione depletion [27]. On the other hand, McCune et al. described only minimal alterations of busulfan CL between doses of i.v. busulfan, with 71.4% of patients having <10% change between doses 1 and 2 [1]. Similar observations were described by Protzenko et al. with no general difference in busulfan CL between the first and the last dose, but marked changes of at least 20% in one third of the patients [23]. A recent publication by Bognar et al. analyzed changes in busulfan CL between day 1 and day 4 in 161 adult patients. They observed a considerable variance in clearance with 78.5% showing a decrease in busulfan CL (median decrease: 3.6%) and 21.5% showing an increase (median increase: 1.6%) [22].

In contrast, our results show no overall decrease in busulfan CL in adult patients receiving once daily intravenous busulfan. The median difference on an individual patient basis between day 1 and day 2 was −0.65% and the proportions of patients exhibiting changes of less than ±10% accounts for 68%. However, one third of patients experience relevant differences in busulfan CL between doses of more than 10% that range from −42 to +58%, reflecting the high variability also reported in other studies [17, 22, 27].

One limitation of our study is that it is a single-center study. On the other hand, the standardized procedure for the application, blood level measurements and performance of the TDM offers advantages. Our center regularly participates successfully in the Busulfan-TDM interlaboratory proficiency testings [20]. In our center, busulfan TDM is performed as part of routine care on the first day of busulfan administration and on subsequent days only if dose deviations of more than 25% are required. This could theoretically result in a bias when analyzing the change in PK parameters. However, due to the high number of patients analyzed here, this appears to be negligible. Since only very few patients required TDM on day 3 (*n* = 13) or day 4 (*n* = 1), we cannot make reliable statements as to whether clearance in patients may change over time. However, other studies also described changes in clearance between only day 1 and day 2 [1, 17, 18]. Furthermore, we have no data on GST polymorphisms for which a possible influence on busulfan CL is discussed [28].

In conclusion, with our real-world data we confirm the high variability of the pharmacokinetic parameters of busulfan administered intravenously once daily. This occurs both between different patients (*inter*-individual) and between doses within a patient (*intra*-individual). The decreased busulfan CL caused by deferasirox, can be compensated by an initial busulfan dose reduction to 75–80%, whereby similar *inter*- or *intra*-individual variability of pharmacokinetic parameters need to be considered. TDM of busulfan is therefore highly recommended to ensure target attainment and optimal clinical outcome.

## Data Availability

Data are available upon data-specific request.
